# Impact of COVID-19 Lockdown on Anthropometric Variables, Blood Pressure, and Glucose and Lipid Profile in Healthy Adults: A before and after Pandemic Lockdown Longitudinal Study

**DOI:** 10.3390/nu14061237

**Published:** 2022-03-15

**Authors:** José Ignacio Ramírez Manent, Bárbara Altisench Jané, Pilar Sanchís Cortés, Carla Busquets-Cortés, Sebastiana Arroyo Bote, Luis Masmiquel Comas, Ángel Arturo López González

**Affiliations:** 1 General Practitioner Department, Balearic Islands Health Service, 07003 Palma, Illes Balears, Spain; jignacioramirez@telefonica.net (J.I.R.M.); lmasmiquel@hsll.es (L.M.C.); 2 Health Institute of the Balearic Islands (IDISBA), Balearic Islands Health Research Institute Foundation, 07003 Palma, Illes Balears, Spain; pilar.sanchis@uib.es (P.S.C.); angarturo@gmail.com (Á.A.L.G.); 3 Chemistry Department, University Balearic Islands, 07003 Palma, Illes Balears, Spain; 4 Faculty of Odontology, University School ADEMA Palma, 07003 Palma, Illes Balears, Spain; cbusquets@eua.edu.es (C.B.-C.); sarroyobote@hotmail.com (S.A.B.); 5 Investigation Group IUNICS, Health Research Institute of the Balearic Islands (IDISBA), 07003 Palma, Illes Balears, Spain

**Keywords:** COVID-19, cardiovascular risk factors, lockdown, disease

## Abstract

In December 2019, 27 cases of pneumonia were reported in Wuhan. In 2020, the causative agent was identified as a virus called SARS-CoV-2. The disease was called “coronavirus disease 2019” (COVID-19) and was determined as a Public Health Emergency. The main measures taken to cope with this included a state of lockdown. The aim of this study was to assess how the unhealthy lifestyles that ensued influenced different parameters. A prospective study was carried out on 6236 workers in a Spanish population between March 2019 and March 2021. Anthropometric, clinical, and analytical measurements were performed, revealing differences in the mean values of anthropometric and clinical parameters before and after lockdown due to the pandemic, namely increased body weight (41.1 ± 9.9–43.1 ± 9.9), BMI (25.1 ± 4.7–25.9 ± 4.7), and percentage of body fat (24.5 ± 9.1–26.9 ± 8.8); higher total cholesterol levels, with a statistically significant increase in LDL levels and a reduction in HDL; and worse glucose levels (90.5 ± 16.4–95.4 ± 15.8). Lockdown can be concluded to have had a negative effect on health parameters in both sexes in all age ranges, causing a worsening of cardiovascular risk factors.

## 1. Introduction

In December 2019, 27 cases of severe pneumonia of unknown cause were reported in the city of Wuhan (Hubei, China), which had in common their appearance in a wholesale market for fish and live animals [1]. On 7 January 2020, the causative agent was identified as a new virus (Coronaviridae), called SARS-CoV-2 [2]. The disease caused by this virus became internationally known as “coronavirus disease 2019” (COVID-19). The most common clinical manifestations were fever, cough, fatigue, and gastrointestinal symptoms. Respiratory or gastrointestinal symptoms could coexist or be found in isolation [3,4,5]. Depending on individual genetics, ethnic origin, age and geographical location, it has been seen that the clinical manifestations and morbidity and mortality from COVID-19 are different [6,7,8]. On 30 January 2020, COVID-19 was determined as a Public Health Emergency of International Importance (ESPII) and later, on 11 March 2020, declared a global pandemic by the WHO [5].

The rapid spread and severity of the COVID-19 pandemic became a threat to public health, with the lack of effective drugs or vaccines at that time leading governments of more than 100 countries to apply strict measures in their efforts to limit and control the spread of the disease [9,10]. Measures such as a lockdown, quarantine, or isolation of their populations were put in place, in such a way that in April 2020 more than a third of the world’s population was under some type of lockdown [11]. In Spain this was established by Royal Decree 463/2020 of March 14, declaring a state of emergency [12].

This state of lockdown had a negative impact on the physical and mental health of the population, with a decrease in physical activity and a significant change in eating patterns at all ages [13,14,15,16,17,18]. Lifestyle modifications and withdrawal from work, university, or school are all related to boredom and were discovered to cause bingeing or loss of appetite [13,19,20]. A decrease in the consumption of fish, seafood, fruit, and vegetables was found, along with [21] a rise in the consumption of salty and sugary snacks (including desserts, sweets, chips, nuts, crackers, popcorn, peanuts, pistachios, sunflower seeds, etc.) [22,23]. There was also a high prevalence of sleep [24,25,26] and physical activity disorders [6], which are related to unbalanced nutritional patterns in adults and adolescents [27,28].

Consequently, there was an increase in weight in the world population range from 11.1% to 72.4% during the lockdown period. In Spain, the weight gain reported by patients themselves ranged between 12.8% and 44% [29]. People who put on weight during the lockdown also had a more sedentary lifestyle most of the time - watching television and doing on-screen leisure activities, using smartphones, the internet, or socializing online. This weight gain related to COVID-19 will cause an increased risk of developing metabolic disorders in the population with a previous diagnosis of disease [30], but also in the population who had not suffered from these disorders beforehand [31]. Moreover, it has been observed that the population with previous pathology has a higher risk of becoming severely ill if infected by the virus [31,32].

Our objective was to evaluate how these unhealthy lifestyles influenced different anthropometric parameters, blood glucose levels, lipid profile, and blood pressure in a sample of 6236 workers in Spain, with the aim that if at some future time a similar situation occurs, we would be able to take adequate preventive measures to reduce its side effects on people’s health and the development of disease.

## 2. Materials and Methods

**Figure 1:**
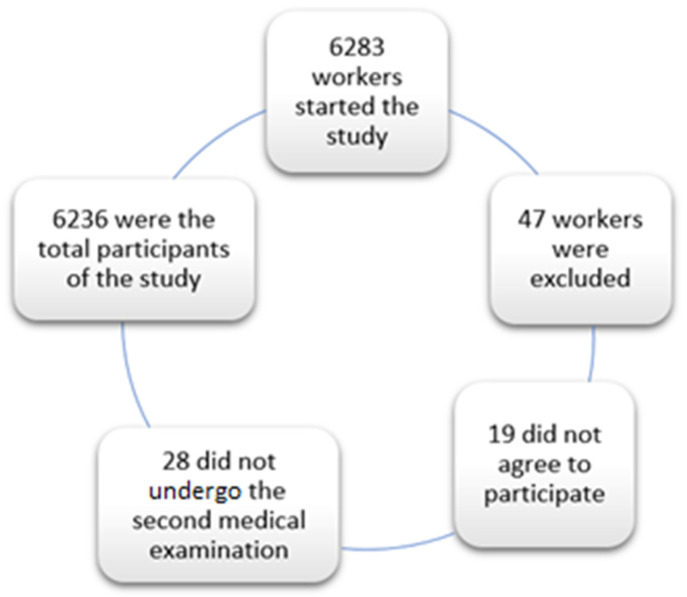
Flowchart of participants.

A prospective study was carried out on 6283 workers in the Balearic Islands and the Valencian Community in companies from different productive sectors, the most represented were working at hotels, construction, commerce, health and public administration, transport, education and the cleaning industry between March 2019 and March 2021. Employees were selected from among those who attended the periodic occupational medical check-ups during those years. Of these, 47 were excluded (19 since they did not agree to participate and 28 since they did not undergo the second medical examination), leaving 6236 finally included in the study (Figure 1).

Inclusion criteria

- Aged between 18 and 69 years;
- Being an active worker;
- Healthy population, without underlying diseases that do not allow passing the annual medical check-up;
- Belonging to one of the companies collaborating in the study;
- Agreeing to participate in the study.

Anthropometric, clinical, and analytical measurements were performed by the health personnel of the different occupational health units participating in the study, after homogenizing the measurement techniques. To measure weight, expressed in kilograms, and height, expressed in cm, a scale with a measuring rod was used, namely model SECA 700 with a capacity of 200 kg and 50-g divisions, with a SECA 220 telescopic measuring rod with millimetric division and a 60–200 cm interval.

Abdominal waist circumference was measured in cm with a measuring tape (SECA model 20, with an interval of 1–200 cm and millimeter division). The person stood feet together and trunk erect, abdomen relaxed, and upper limbs hanging on both sides of the body. The tape measure was placed parallel to the floor at the level of the last floating rib. Hip circumference was measured with a SECA model 200 tape with a measuring interval of 12–200 cm and millimeter division. The same position was adopted as for waist circumference and the measuring tape was passed horizontally at hip level. Waist/height and waist/hip indices were obtained by dividing waist circumference by height and hip circumference, respectively. The cut-off point for the former was 0.50 while for the latter it was 0.85 for females and 0.95 for males [33].

Blood pressure was measured in the supine position with a calibrated OMRON M3 automatic sphygmomanometer after 10 min of rest. Three measurements were taken at one-minute intervals and the mean of the three was calculated. Blood tests were obtained by peripheral venepuncture after a 12-h fast, sent to reference laboratories, and processed within 48–72 h. Automated enzymatic methods were used for blood glucose, total cholesterol, and triglycerides. Values are expressed in mg/dL. HDL was determined by precipitation with dextran sulphate Cl2Mg, and values are expressed in mg/dL. LDL was calculated using the Friedewald formula (provided triglycerides were less than 400 mg/dL). Values are expressed in mg/dL.

Friedewald’s formula:

LDL = total cholesterol − HDL − triglycerides/5

BMI was calculated by dividing weight by height in meters squared. Obesity was considered to be over 30 [33]. Body fat percentage was determined by bioimpedance using a Tanita model MC-780MA S.

A smoker was considered to be a person who had regularly consumed at least one cigarette/day (or the equivalent in other types of consumption) in the previous month or had stopped smoking less than a year before.

Physical activity was determined by means of the International Physical Activity Questionnaire (IPAQ) [34], a seven-question self-administered questionnaire that assesses the type of physical activity performed in daily life during the previous seven days which was performed at each medical check-up.

### 2.1. Statistical Analysis

A descriptive analysis of the categorical variables was carried out by calculating the frequency and distribution of responses for each one. For quantitative variables, the mean and standard deviation were calculated, whereas for qualitative variables, the percentage was calculated. Bivariate association analysis was performed using the X^2^ test (with correction of Fisher’s exact statistic when conditions so required) and Student’s *t* test for independent samples. For multivariate analysis, binary logistic regression was used with the Wald method, with calculation of the odds ratio and the Hosmer-Lemeshow goodness-of-fit test. The statistical analysis was performed with the SPSS 27.0 (IBM, New York, USA) program, with an accepted statistical significance level of 0.05.

### 2.2. Ethical Considerations and Aspects

The study was approved by the Clinical Research Ethics Committee of the Balearic Islands Health Area no. IB 4383/20. All procedures were performed in accordance with the ethical standards of the institutional research committee and with the 2013 Declaration of Helsinki. All patients signed written informed consent documents before participating in the study.

## 3. Results

Lockdown began for all participants on 15 March 2020, and post-lockdown anthropometric measurements were carried out by the same health personnel from the different occupational health units. Of the 6283 workers, 51.9% were female and 48.1% were male, constituting a proportional representation of both sexes. Participant characteristics, including anthropometric characteristics, physical activity, and smoking before and after lockdown, are all summarized in Table 1. The number of participants was the same each year, being a total of 6236 Spanish workers.

Table 1 shows the statistically significant differences in the mean values of anthropometric and clinical parameters before and after lockdown due to the COVID-19 pandemic.

An increase in body weight and therefore also in BMI can be seen in the population studied; as well as an increase in the percentage of body fat, and hip and waist circumference; being this the one with the highest increase. Regarding clinical parameters, the elevation of total cholesterol levels stands out, with a statistically significant increase in LDL levels (going from mean values of 117.4 mg/dL to 131 mg/dL) and a reduction in HDL levels. Triglyceride values also rose.

Comparing blood pressure levels, during lockdown there was a tendency to higher diastolic blood pressure levels. Systolic blood pressure was also affected. Glucose levels increased during lockdown such as the previously analyzed parameters.

The percentage of people who increased their smoking habit during lockdown was 2%, while an 11% decrease their physical activity, causing an elevation of 4.1% in overweight and 2.5% in obesity.

Comparing the mean values of the anthropometric, clinical, and laboratory parameters in different ranges of years according to whether this change occurred before or during the COVID-19 pandemic lockdown, it is possible to observe a tendency to a worsening of the values of the different parameters analyzed, as shown in Table 2.

**Figure 2:**
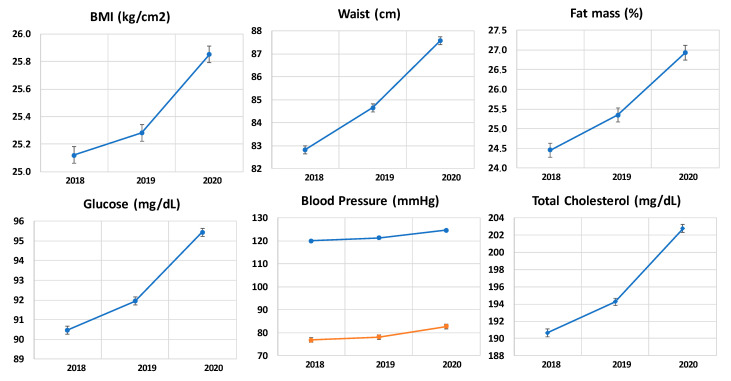
Changes in cardiovascular risk factors in 2018, 2019, and 2020: BMI, waist, lean mass, glucose, blood pressure, and total cholesterol.

Figure 2 shows the graphs of the parameters related to cardiovascular risk factors. It can be observed that over the years, the trend in all of them was towards an increase in mean values, with an exponential, statistically significant increase during the year of the pandemic due to COVID-19. It can be seen an increase in BMI levels, percentage of fat mass, laboratory values of glucose and total cholesterol, as well as higher blood pressure levels. The alteration encountered in mean blood pressure values was less pronounced compared to the rest of the parameters analyzed, which underwent greater changes, causing an increase in cardiovascular risk in the population studied.

**Figure 3:**
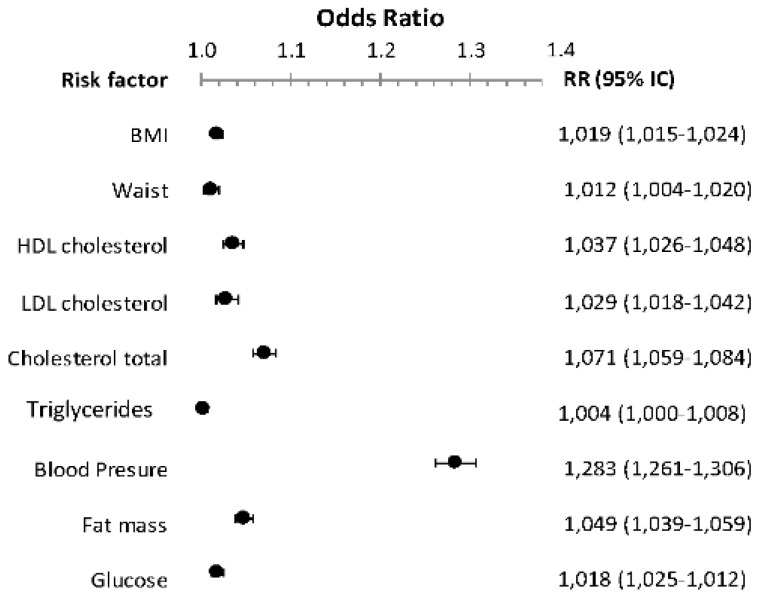
Relative Risk (RR) of patients who had worsened cardiovascular risk factors in the 2019–2020 season when compared with the 2018–2019 season. Patients were considered to have worsened when they changed to a worse category in the corresponding risk factor: BMI (normal, overweight, obesity); glycemic status (normal, prediabetes, diabetes); blood pressure status (normal, pre-AHT, AHT1, AHT2); waist (normal, high); fat mass (normal, high); total cholesterol (normal, high); LDL (normal, high, very high); HDL (normal, low); and TG (normal, high).

Comparing the different cardiovascular risk parameters that had worse mean values after COVID (and considering an increased risk when the category changed to a worse one according to the corresponding risk factor), it can be observed in Figure 3 that by analyzing the parameters according to relative risk, with a 95% confidence interval, and odds ratio, the changes in the mean values of triglyceride levels were not related to the other parameters analyzed. In the other variables analyzed, blood pressure levels were those with the highest RR (1.261–1.306) along with OR (1.283). The rest of the parameters can be seen in the figure.

The parameters of BMI, glycemic status, blood pressure, waist circumference, and percentage of fat mass were analyzed in relation to age, sex, BMI, glycemic status, and blood pressure levels. It can be observed in Table 3 that upon analyzing by age variable, there is a statistically significant worsening of the values of BMI, glycemic status, blood pressure, and waist circumference, which does not occur in the percentage of fat mass, although this relationship is not statistically significant with age. In the group over 50 years of age, the RR is higher.

When analyzing by sex, the BMI values obtained are not considered statistically significant, although there is statistical significance for the rest of the variables studied. It should be noted that, in males, there is no causal relationship with an increase in percentage of fat mass, while in females there is.

There is no clear association between overweight or obesity and glucose levels, with a RR < 1 and a *p*-value of 0.241. In terms of BMI, despite having a causal relationship with the percentage of fat mass with a RR > 1, the values obtained have a *p*-value of 0.704, therefore this relationship and the levels obtained could be caused by other factors as they are not statistically significant. Changes in BMI are not always related to a higher percentage of fat mass; patients with high muscle mass and a low percentage of fat mass, for instance, could also have altered BMI values since this parameter does not differentiate between muscle or fat.

By analyzing glycemic status, workers with prediabetes did reveal a direct relationship with alterations in percentage of fat mass, but not with blood pressure levels or waist circumference. When relating baseline blood glucose levels to BMI, despite observing a causal relationship with a RR > 1, the values obtained were not statistically significant (*p*-value 0.065) and would therefore not be interpretable for this reason.

Blood pressure levels do not have a statistically significant relationship with glycemic status. Although the relationship between blood pressure and waist circumference and percentage of fat mass have been statistically related to a *p*-value < 0.001, in the group of workers with type 1 hypertension, there was no increase in blood glucose levels as there was no direct association with the anthropometric parameters analyzed.

When analyzing according to age of participants, a statistically significant increase in total cholesterol levels was observed, with a greater association in workers aged 40–50 years) with a *p*-value of < 0.001. There are also statistically significant differences with *p*-value < 0.001 in LDL cholesterol levels with a lower association in workers over 50 years old), as can be seen in Table 4.

When the relationship between age, HDL cholesterol, and triglyceride levels is analyzed, the differences are not statistically significant with a *p*-value of > 0.05, so the changes in the parameters could be due to other factors and not by age.

According to the sex of the patient studied, a statistically significant worsening stands out with a direct association for the clinical parameters of total cholesterol, HDL cholesterol, and LDL cholesterol in both males and females, but not for triglyceride levels where the differences between males) and females are not statistically significant.

In the population studied that was overweight or obese, the increased analytical values of total cholesterol and the decrease in HDL cholesterol were statistically significant (*p*-value < 0.05) even though in the group of overweight patients, there was no direct association with HDL cholesterol values. No statistically significant relationship was found with LDL cholesterol or triglyceride levels in the overweight and obese population.

The glycemic level of the workers studied was found to have a direct, statistically significant association with a *p*-value of < 0.05 for HDL cholesterol and LDL cholesterol, but not with total cholesterol or triglyceride levels. In the group of patients with prediabetes, no direct relationship with changes in LDL cholesterol was observed.

If we analyze according to blood pressure levels, patients with normal blood pressure or type II hypertension are associated with a higher risk (RR: 1.106; 95% CI 1.081–1.132) and (RR: 1.053; 95% CI 0.620–1.787) for total cholesterol and a RR: 1.005; 95% CI 0.987–1.024 and RR: 1.003; 95% CI 0.955–1.053 for HDL cholesterol, respectively; but with a non-statistically significant association for LDL cholesterol levels with a *p*-value of 0.072.

Regarding triglyceride levels, according to blood pressure levels, no direct relationship was found in patients with hypertension, but was found in normotensive patients with a *p*-value of 0.036.

In relation to physical activity, the changes caused by lockdown, measured through the IPAQ questionnaire, can be seen in Table 5. A decrease in physical activity is observed in both sexes for the group that exercised before lockdown, as well as an increase in sedentary lifestyle in groups that did not perform physical activity before lockdown on a regular basis.

The decrease in physical activity is statistically significant for both sexes, with a *p*-value < 0.0001 compared to the time before the pandemic.

## 4. Discussion

The global pandemic caused by COVID-19 has had a great impact on health population [35]. Not only due to the infection caused by the virus, which has left complications and consequences of the disease from which some people are yet to fully recover, but also due to the pathology derived from lockdown, social distancing, and isolation, in which chronic diseases have worsened [36,37,38,39]. These measures taken by governments to protect public health have produced a psychological impact on the population that has caused overeating, a more sedentary lifestyle, and modification of several anthropometric, clinical, and laboratory health parameters affecting all body systems [40,41].

In this study, we objectify the changes produced in the population due to lockdown, in a population of Spanish workers. Although it is true that, in recent decades, the population has had a tendency to obesity and overweight due to a sedentary lifestyle [13,16], what can be seen in this study is that, during lockdown, many of the parameters that influence cardiovascular risk were affected, such as obesity, alcoholism, and a sedentary lifestyle, changing their values, leading to a greater risk of suffering from cardiovascular diseases. Our results are similar to those published by other authors from different countries such as Lithuania, China, Korea, Israel, UK, amongst others [40,41,42,43,44,45].

In the study carried out by Paltrienteri et al., the changes produced during lockdown in relation to physical activity, diet, alcohol consumption, and tobacco were studied through a self-administered survey. The results showed there had been a reduction in physical activity without a change in diet [46]. In our study, these modifications were studied through anthropometric, clinical, and laboratory parameters, and their changes could be observed with a statistically significant increase in both obesity and overweight, as seen in our results.

A simultaneous to the increase in cardiovascular risk, as detailed in Table 1, the 4.1% increase in the rate of overweight and obesity, as well as the 11% decrease in physical activity and 2% increase in smoking brought about the development of other diseases, both acute and chronic, which have modified the health status of the population and increased morbidity and mortality from other causes, not only due to infection with COVID-19. These consequences were also pointed out by Palmer et al. [47,48].

The results of our study reveal statistically significant differences when comparing clinical, laboratory, and anthropometric parameters in a population of workers due to a lockdown. The increase in body weight and therefore BMI is a consequence of the dietary habits and sedentary lifestyle of the population during this period [49,50]. An increase in percentage of fat mass and waist and abdominal perimeter was also observed [42,44,45]. There are studies in the literature that compare body weight during and after lockdown, such as the study by Blautani S, et al. which shows that it was not the entire population that suffered an increase in body weight, with 18.2% of the population in their study losing weight during lockdown. At the end of lockdown, those who had gained weight continued to gain, so it is likely that their health effects associated to body changes will persist over time [51] if lifestyle modifications are not made [52].

Not only were anthropometric parameters affected, but alterations were also found in biochemical parameters. In the lipid profile, for instance, an increase in total cholesterol levels was detected, which corresponded to an additional increase in LDL cholesterol levels and decrease in HDL cholesterol levels, with statistically significant differences. These results are similar to those published in other studies, although their sample sizes were much smaller than ours [40,53].

Plasma glucose levels deteriorated, probably in connection with the increased rate of obesity, overweight, and decrease in physical exercise [54]. In patients with blood glucose levels in the range of diabetes mellitus, a statistically significant decrease of LDL cholesterol levels was detected, although this was not statistically significant in patients with prediabetes values, in whom there was no clear relationship with changes in LDL cholesterol values. At a general level, there was an increase of triglyceride levels with statistically significant results. The combined increase in serum triglycerides points to the role of a variation in eating habits and reinforces the need not to attenuate attention to an adequate lifestyle program, with regular physical activity and a correct dietary approach, even when successful pharmacological treatment is ongoing [54]. A high-carbohydrate diet is known to raise fasting triglyceride levels more than a high-fat diet, which is also related to greater mortality [55]. Our results are similar to those obtained in previous studies [40,53].

Regarding blood pressure levels, lockdown also caused a deterioration in people who were not previously hypertensive, probably due to their lifestyle during these months and the worsening of the population’s health status owing to a change in dietary habits and physical activity. In the literature consulted, we have found very few studies that refer to changes in blood pressure during lockdown due to COVID-19. However, the studies published are similar to our results [40,56].

The lockdown adversely affected multiple risk factors for disease, especially cardiovascular disease. Plasma concentrations for LDL and HDL cholesterol, respectively increased and decreased. Concurrently, blood glucose concentrations and blood pressures increased. According to the increases in ratio of waist to hip circumferences, these effects were associated with increased central obesity [57]. Notably, low HDL cholesterol, a large waist circumference, hyperglycemia, hypertension, and hypertriglyceridemia are components of metabolic syndrome, a global measure of risk for cardiovascular disease and developing type 2 diabetes mellitus [58]. According to the latest guidelines, the metabolic syndrome is described as a set of analytical and anthropometric alterations, in which the patient must have at least three altered parameters in order to be diagnosed of metabolic syndrome. These parameters are: waist circumference in men ≥102 cm and ≥88 cm in women, triglyceride values ≥150 mg/dL or being on pharmacological treatment, HDL levels <40 mg/dL in men and <50 mg/dL in women. Blood pressure values ≥130/85 mmHg or being on pharmacological treatment with antihypertensives and fasting blood glucose levels ≥100 mg/dL or being on antihyperglycemic treatment [59].

According to the results obtained in our study, which have been explained previously, a global worsening of these parameters could be detected, secondary to the change in lifestyle caused by lockdown. The increased metabolic syndrome has been able to develop the appearance of different cardiovascular and metabolic complications that have caused an increase in morbidity and mortality, as well as an increased risk of COVID-19 infection with greater potential for severity as has been seen in recent studies [60,61,62] as shown in the study carried out by Li B et al., which shows how the population with cardiovascular risk factors has a higher risk of severe infection by COVID-19 [63].

Periodic medical check-ups, in the case of our study, of Spanish workers, has allowed the detection of these alterations and the possibility of applying preventive measures to avoid the development of diseases in the future as well as complications in the event of infection by COVID-19 [60,61].

In the different tables of results, the worsening of blood pressure levels, glycaemia, waist-abdominal perimeter as well as analytical levels of triglycerides and HDL cholesterol are observed. Early application of preventive measures in the different altered parameters could prevent the development of metabolic syndrome and its possible complications [59,62,64].

Despite being known as a syndrome, there is no single approach, since the objective is to apply preventive or therapeutic measures individually according to the altered parameters [65,66] in each individual, always starting with lifestyle modifications, a factor that has been greatly influenced by lockdown due to the increase in sedentary lifestyle, with a decrease in physical activity [67,68,69] and the complications driven by COVID-19.

The aim of treating or preventing these altered analytical and anthropometric parameters produced by the state of lockdown would be to prevent the development of cardiovascular diseases and therefore reduce cardiovascular risk at the population and its potential morbidity and mortality. In the study conducted by Yangjing X, et al., people with established cardiovascular disease or altered cardiovascular risk parameters are shown to have a worse prognosis when faced with COVID-19 infection [70].

In our study, many of these cardiovascular risks are seen to have modified their levels during lockdown, leading to an increase in cardiovascular risk. Also, the fact of decreasing physical activity and increasing sedentary lifestyle is associated with an increase and worsening in the different parameters studied and also with a tendency to obesity and therefore to the complications derived from it, causing an increase in cardiovascular risk factors in the population [71], as has also been seen in other studies such as the ones from Hendren et al. or Hu L et al. [72,73].

In our study, we found a significant decrease in physical activity both in men and women, being higher in women. Our results are consistent with other published studies [74,75], and differ from those obtained by Castañeda-Barbarro et al.’s. in which a greater decrease in physical exercise in men is found [76].

Regular physical activity helps reduce cardiovascular risk [68], by reducing the percentage of fat mass and improving laboratory and clinical values such as blood pressure, insulin resistance… helping in the prevention of developing diseases derived from an unhealthy lifestyle and preventing cardiovascular diseases [69,77].

With all this, it can be stated that the COVID-19 pandemic has increased the risk of developing pathologies derived from lifestyle modifications, in addition to raising the risk of COVID-19 infection by altering parameters that increase the risk of illness [54,69,78].

## 5. Strengths and Limitations

Several studies compare the effects of the pandemic and COVID-19 infection with changes in obesity and overweight parameters, as well as cardiovascular risk factors and other pathologies, but we have not found any study in which so many parameters are compared in the same population as in our study.

Further, none of the studies that present the evaluation of the different parameters separately have a sample size such as ours, with 6283 patients.

The limitations found in this study are the fact that it was carried out in a specific geographic area, with a Caucasian working population, over a certain period of time, which could limit the generalization of the results to other areas where lifestyles may be different.

Selection bias is another limitation of our study, since it is limited to workers who voluntarily attended company medical examinations during those years.

Therefore, the results do not apply to other populations, and specific studies would have to be carried out.

## 6. Conclusions

Health behaviors have been negatively affected during lockdown, leading to an increase in sedentary behavior in all age groups, an unhealthy diet, and, therefore, associated with weight gain, as well as an increased consumption of tobacco.

With the parameters and results described above, it can be concluded that the months of lockdown caused a statistically significant deterioration of several health parameters due to increased sedentary behavior in a similar way in males and females in all age ranges, although the over 40-year-old group is the one where the worst values of the variables analyzed were observed, causing an increase in the magnitude of multiple risk factors for cardiovascular disease and the appearance of new pathologies that have resulted in an increase in morbidity and mortality due to all causes.

## Figures and Tables

**Table 1:** Characteristics of the population.

	Year 2018	Year 2019	Year 2020	
N = 6236	Mean ± SD	Mean ± SD	Mean ± SD	*p*-Value
Age (years)	41.1 ± 9.9	42.1 ± 9.9	43.1 ± 9.9	<0.001
Weight (kg)	71.7 ± 16.3	72.2 ± 16.4	73.8 ± 16.5	<0.001
BMI (kg/m^2^)	25.1 ± 4.7	25.3 ± 4,7	25.9 ± 4.7	<0.001
Waist circumference (cm)	82.8 ± 14.0	84.6 ± 14.1	87.6 ± 14.1	<0.001
Hip circumference (cm)	98.7 ± 9.4	99.8 ± 9.4	101.5 ± 9.5	<0.001
Waist to Height ratio	0.49 ± 0.08	0.50 ± 0.08	0.52 ± 0.08	<0.001
Waist to hip ratio	0.84 ± 0.10	0.85 ± 0.09	0.86 ± 0.09	<0.001
Body fat (%)	24.5 ± 9.1	25.3 ± 8.7	26.9 ± 8.8	<0.001
SBP (mmHg)	120.0 ± 16.8	121.3 ± 16.3	124.6 ± 16.3	<0.001
DBP (mmHg)	76.9 ± 10.7	78.2 ± 10.5	82.8 ± 10.6	<0.001
Glycaemia (mg/dL)	90.5 ± 16.4	91.9 ± 15.7	95.4 ± 15.8	<0.001
Total cholesterol (mg/dL)	190.7 ± 37.3	194.3 ± 35.3	202.8 ± 35.7	<0.001
HDL-c (mg/dL)	53.9 ± 13.7	53.1 ± 13.4	50.7 ± 13.7	<0.001
LDL-c (mg/dL)	117.4 ± 40.3	121.4 ± 38.5	131.0 ± 39.0	<0.001
Triglycerides (mg/dL)	96.8 ± 79.2	98.7 ± 78.5	105.8 ± 78.9	<0.001
	N (%)	N (%)	N (%)	*p*-value
Smokers	1176 (18.9)	1202 (19.3)	1302 (20.9)	<0.001
Physical exercise	2732 (43.8)	2600 (41.7)	2044 (32.8)	<0.001
Normal weight	3500 (56.1)	3398 (54.5)	3085 (49.5)	<0.001
Overweight	1890 (30.3)	1978 (31.7)	2144 (34.4)	
Obesity	846 (13.6)	860 (13.8)	1007 (16.1)	
Waist to height ratio high	2526 (40.5)	2826 (45.3)	3368 (54.0)	<0.001
Waist to hip ratio high	1460 (23.4)	1612 (25.8)	1944 (31.2)	<0.001
Body fat normal	4115 (66.0)	3996 (64.1)	3722 (59.7)	<0.001
Body fat high	1394 (22.4)	1428 (22.9)	1466 (23.5)	

SBP: systolic blood pressure; DBP: diastolic blood pressure; HDL: high density lipoproteins; LDL: low density lipoproteins.

**Table 2:** Changes in anthropometric, clinical, laboratory, and healthy habit variables in different pre-COVID and COVID years.

	2018–2019 Change	2019–2020 Change
Weight (kg)	0.47 ± 1.04	1.61 ± 1.28
BMI (kg/m^2^)	0.16 ± 0.37	0.57 ± 0.46
Waist circumference (cm)	1.82 ± 4.87	2.92 ± 1.17
Hip circumference (cm)	1.14 ± 0.85	1.69 ± 1.15
Waist to Height ratio	0.01 ± 0.03	0.02 ± 0.01
Waist to hip ratio	0.01 ± 0.05	0.01 ± 0.02
Body fat (%)	0.88 ± 2.14	1.58 ± 1.68
SBP (mmHg)	1.28 ± 4.08	3.26 ± 3.68
DBP (mmHg)	1.35 ± 1.48	4.62 ± 1.82
Glycaemia (mg/dL)	1.47 ± 5.16	3.49 ± 2.30
Total cholesterol (mg/dL)	3.59 ± 17.30	8.52 ± 13.40
HDL-c (mg/dL)	−0.82 ± 3.97	−2.44 ± 1.78
LDL-c (mg/dL)	4.04 ± 17.64	9.54 ± 13.41
Triglycerides (mg/dL)	1.83 ± 8.74	7.09 ± 4.63

**Table 3:** Relative risk of patients with increased BMI, waist, fat mass, glycemic status, and hypertension status divided into categories of age, sex, BMI, glycemic status, and hypertension status. Patients were considered to have worsened when they changed to an upper category in the corresponding risk factor: BMI (normal, overweight, obesity); glycemic status (normal, prediabetes, diabetes); blood pressure status (normal, pre-AHT, AHT1, AHT2); waist (normal, high); fat mass (normal, high, very high); total cholesterol (normal, high); LDL (normal, high); HDL (normal, low); TG (normal, high).

		BMI			Glucemic Status			Blood Pressure			Waist			Fat Mass		
		RR	(95% CI)	*p*-Value of Interaction	RR	(95% CI)	*p*-Value of Interaction	RR	(95% CI)	*p*-Value of Interaction	RR	(95% CI)	*p*-Value of Interaction	RR	(95% CI)	*p*-Value of Interaction
Age				<0.001			<0.001			<0.001			<0.001			0.907
	<35	1.008	(1.001–1.014)		1.010	(1.001–1.019)		1.223	(1.186–1.261)		1.009	(0.997–1.020)		1.084	(1.062–1.106)	
	35–40	1.021	(1.012 1.030)		1.021	(1.008–1.035)		1.287	(1.234–1.343)		1.013	(1.000–1.027)		1.037	(1.015–1.060)	
	40–50	1.020	(1.013–1.028)		1.022	(1.011–1.034)		1.323	(1.283–1.365)		1.017	(1.004–1.029)		1.022	(1.007–1.037)	
	>50	1.029	(1.018–1.041)		1.021	(1.004–1.038)		1.284	(1.235–1.336)		0.999	(0.975–1.023)		1.064	(1.042–1.087)	
Gender				0.453			<0.001			<0.001			<0.001			<0.001
	man	1.024	(1.017–1.031)		1.022	(1.011–1.033)		1.372	(1.335–1.411)		1.002	(0.991–1.013)		0.971	(0.957–0.985)	
	women	1.015	(1.010–1.021)		1.014	(1.007–1.021)		1.212	(1.186–1.238)		1.022	(1.011–1.032)		1.125	(1.112–1.139)	
BMI							0.241			<0.001			<0.001			0.704
	Normal	-			0.986	(1.007–1.021		1.230	(1.204–1.257)		1.008	(1.004–1.011)		1.075	(1.061–1.089)	
	Overweight	-			0.998	(0.980–1.024)		1.293	(1.252–1.335)		1.044	(1.022–1.066)		1.009	(0.993–1.025)	
	Obesity	-			0.968	(1.019–1.045)		1.516	(1.431–1.606)		0.954	(0.927–0.982)		1.038	(1.014–1.063)	
Glucemic status				0.065			0.004			0.001			0.235			0.013
	NORMAL	1.015	(1.011–1.020)			(-)		1.061	(1.050–1.073)		1.017	(1.009–1.025)		1.029	(1.016–1.043)	
	prediabetes	1.041	(1.027–1.055)			(-)		0.989	(0.967–1.011)		0.977	(0.954–1.002)		1.028	(1.001–1.056)	
	diabetes	1.031	(0.996–1.067)			(-)		0.993	(0.934–1.056)		1.050	(0.978–1.129)		1.070	(0.979–1.170)	
Blood Pressure				<0.001			0.280						<0.001			<0.001
	normal	1.011	(1.006 1.017)		1.015	(1.004 1.025			(-)		1.018	(1.005 1.031)		1.102	1.085 1.119	
	preHTA	1.023	(1.016–1.030)		1.016	(1.007–1.025)			(-)		1.016	(1.005–1.028)		1.033	(1.019–1.048)	
	HTA 1	1.019	(1.007–1.030)		1.021	(1.004–1.038)			(-)		0.990	(0.972–1.009)		0.994	(0.970–1.019)	
	HTA 2	1.033	(1.008–1.059)		1.042	(1.010–1.073)			(-)		1.002	(0.964–1.042)		1.026	(0.983–1.072)	

**Table 4:** Relative Risk of patients with increased total cholesterol, LDL, TG and a decrease in HDL levels, divided by categories of age, sex, BMI, glycemic status and hypertension status. Patients were deemed to have worsened when they changed to an upper category in the corresponding risk factor: BMI (normal, overweight, obesity); glycemic status (normal, prediabetes, diabetes); blood pressure status (normal, pre-AHT, AHT1, AHT2); waist (normal, high); fat mass (normal, high, very high); total cholesterol (normal, high); LDL (normal, high); HDL (normal, low); and TG (normal, high).

		Total Cholesterol			HDL			LDL			TG		
		RR	(95% CI)	*p*-Value of Interaction	RR	(95% CI)	*p*-Value of Interaction	RR	(95% CI)	*p*-Value of Interaction	RR	(95% CI)	*p*-Value of Interaction
Age				0.001			0.958			<0.001			0.902
	<35	1.049	(1.020–1.139)		1.047	(1.027–1.068)		1.047	(1.025–1.069)		1.012	(1.004–1.021)	
	35–40	1.049	(1.021–1.078)		1.028	(0.998–1.059)		1.017	(0.991–1.044)		1.004	(0.994–1.015)	
	40–50	1.089	(1.066–1.113)		1.028	(1.011–1.046)		1.048	(1.026–1.069)		0.999	(0.994–1.004)	
	>50	1.008	(0.982–1.035)		1.044	(1.022–1.068)		0.982	(0.955–1.011)		1.000	(0.990–1.010)	
Gender				<0.001			0.000			0.040			0.643
	Men	1.061	(1.044–1.080)		1.078	(1.059–1.097)		1.018	(1.001–1.035)		0.999	(0.993–1.006)	
	Women	1.081	(1.062–1.099)		1.002	(0.990–1.014)		1.041	(1.024–1.058)		1.008	(1.003–1.012)	
BMI category				0.027			0.000			0.327			0.220
	Normal	1.092	(1.074–1.110)		1.046	(1.033–1.060)		1.040	(1.024–1.057)		1.004	(0.999–1.010)	
	Overweight	1.049	(1.027–1.071)		0.989	(0.958–1.021)		1.011	(0.979–1.045)		1.006	(0.999–1.013)	
	Obesity	1.041	(1.007–1.075)		1.038	(1.017–1.060)		1.011	(0.979–1.045)		0.995	(0.984–1.006)	
Glucemic status				0.113			0.015			0.003			0.596
	Normal	1.066	(1.052–1.080)		1.004	(1.000–1.009)		1.277	(1.253–1.301)		1.034	(1.023–1.046)	
	prediabetes	1.111	(1.079–1.143)		0.996	(0.987–1.006)		1.299	(1.237–1.365)		1.066	(1.033–1.099)	
	diabetes	1.002	(0.932–1.077)		1.042	(1.001–1.084)		1.506	(1.272–1.783)		0.866	(0.774–0.969)	
Blood Pressure				<0.001			0.006			0.072			0.036
	normal	1.106	(1.081–1.132)		1.005	0.987 1.024		1.040	(1.018 1.062)		1.004	0.996 1.013	
	preHTA	0.545	(0.464–0.641)		1.055	(1.039–1.071)		1.026	(1.009–1.044)		1.007	(1.001–1.012)	
	HTA 1	0.639	(0.478–0.853)		1.059	(1.030–1.089)		1.022	(0.992–1.053)		0.997	(0.990–1.004)	
	HTA 2	1.053	(0.620–1.787)		1.003	(0.955–1.053)		1.023	(0.979–1.069)		0.999	(0.981–1.017)	

**Table 5:** Percentage of physical activity, before and during lockdown in men and women separated into groups according to whether or not they previously performed physical activity.

	Year 2018	Year 2019	Year 2020	*p*-Value
Women non physical exercise	57.0	57.4	69.2	<0.0001
Women yes physical exercise	43.0	42.6	30.8	
Men non physical exercise	55.3	59.3	65.1	<0.0001
Men yes physical exercise	44.7	40.7	34.9	

## Data Availability

Data available on request due to restrictions (privacy or ethical).
